# Prevalence of presenting bilateral visual impairment associated with refractive error – findings from the See4School, pre-school vision screening programme in NHS Scotland

**DOI:** 10.1038/s41433-024-03047-8

**Published:** 2024-04-10

**Authors:** Lee Pentland, Miriam Louise Conway

**Affiliations:** 1 Ninewells Hospital Tayside, Dundee, UK; 2 City, University of London, London, UK

**Keywords:** Quality of life, Health care

## Abstract

**Background/objectives:**

The See4School programme in Scotland is a pre-school vision screening initiative delivered by orthoptists on a national scale. The primary objective of any vision screening programme is to identify amblyopia, given the common understanding that this condition is unlikely to be detected either at home or through conventional healthcare channels. The target condition is not bilateral visual impairment, as it is believed that most children will be identified within the first year of life either through observations at home or as part of the diagnosis of another related disorder. This belief persists even though bilateral visual impairment is likely to have a more detrimental impact on a child’s day-to-day life, including their education. If this hypothesis were accurate, the occurrence of bilateral visual impairment detected through the Scottish vision screening programme would be minimal as children already under the hospital eye service are not invited for testing. The overarching aim of this study was therefore to determine the prevalence of presenting bilateral visual impairment associated with refractive error detected via the Scottish preschool screening programme.

**Subjects/methods:**

Retrospective anonymised data from vision screening referrals in Scotland from 2013–2016 were collected. Children underwent an assessment using a crowded logMAR vision test and a small number of orthoptic tests.

**Results:**

During the 3-year period, out of 165,489 eligible children, 141,237 (85.35%) received the vision screening assessment. Among them, 27,010 (19.12%) failed at least one part of the screening and were subsequently referred into the diagnostic pathway, where they received a full sight test. The prevalence of bilateral visual impairment associated with refractive error and detected via the vision screening programme (≥ 0.3LogMAR) was reported to range between 1.47% (1.37–1.59) and 2.42% (2.29–2.57).

**Conclusions:**

It is estimated that up to 2.42% (2.29–2.57) of children living Scotland have poorer than driving standard of vision (6/12) in their pre-school year, primarily due to undetected refractive error. Reduced vision has the potential to impact a child’s their day-to-day life including their future educational, health and social outcomes.

## Introduction

In 1979, the World Health Organization (WHO) defined blindness as a measure of an individual’s best corrected visual acuity [1]. This classification excluded individuals whose day-to-day life was impacted by uncorrected refractive error. In more recent years it has been acknowledged that the classification for visual impairment is too limited and that it should be amended to include uncorrected refractive error or correctable visual impairment [2]. Consequently, the WHO has revised its definition from a classification based on “best corrected visual acuity” to one based on “presenting visual acuity” [3].

In October 2022, the World Health Organisation (WHO) published a factsheet [3] recognising that uncorrected refractive error is one of the top 5 leading causes of global visual impairment. According to this factsheet, the estimated the prevalence of global blindness stands at 2.2 billion, of which 88.4 million having moderate or severe visual impairment (MSVI) due to unaddressed refractive error (excluding presbyopia). This figure represents an increase from previous estimates published in the Lancet in 2021 [4], when the estimate was approximately 86.1 million. Notably, a Cochrane report by Evans et al. published in 2018 [5] found that uncorrected refractive error is the leading cause for reduced vision in children in the UK.

To address persistent worldwide inequalities in access to eye care services, the WHO has developed a package of interventions [6]. The primary objective of this package is to enable countries to identify and incorporate critical eye care interventions into universal health coverage [6]. Within this framework, twelve high-quality clinical guidelines applicable to refractive error were identified [7], with vision screening for all children aged between 3 to 5 years consistently recommended to detect significant strabismus, refractive errors and amblyopia.

In 2019, the United Kingdom’s National Screening Committee (NSC) was tasked with reviewing new research evidence which address gaps in the literature, including the clinical effectiveness of vision screening in children aged 4 to 5 years old. The NSC focused their review on the adverse impacts associated with amblyopia, as this was the target condition from earlier reviews and because they believed that the majority of children with visual impairment in both eyes are detected during their first year of life [8]. The negative impacts related to bilateral refractive error or manifest strabismus were not included in their review, and the authors concluded that there was an absence of direct evidence for the clinical effectiveness of screening.

Global evidence suggests that parents might not always be aware of when a child is struggling to see clearly [9, 10], particularly if visual impairment is mild and the child’s vision is still developing. Younger children often face challenges expressing themselves and may struggle to articulate their symptoms as clearly as adults. Cross sectional study data from the United Kingdom indicates that children with milder impairments often fail to access eye care [11]. The study reported that 9% of four to five-year-old children from a vision screening programme in Bradford, had a presenting visual acuity of >0.2logMAR. In addition, the study reported that decreased visual acuity was associated with reduced literacy, potentially influencing their future educational, health and social outcomes [11]. These figures are likely exacerbated among children living in households with more deprived backgrounds [12], or certain ethnicities, where there are higher levels of reported barriers to eye care [13]. Emotional and behavioural problems are common among young children with significant visual impairment [14]. In a qualitative evaluation conducted by Dudovitz et al. [15] within a school-based vision programme, corrective lenses were found to improve focus, class participation, effort, task persistence, and completion of homework.

Since 2012, Scotland has implemented a national vision screening programme called See4School. This programme, delivered by orthoptists aims to screen the entire population of preschool children. In majority of Health Boards (HB’s), orthoptists conduct the screening within the nursery setting. Children who are not seen in their nursery, are offered appointments at community or hospital clinics. The purpose of the service is early detection of refractive error, amblyopia, strabismus and binocular vision defects, all of which can have a detrimental impact on a children’s literacy [15–19]. The consensus is well-established that amblyopia stands as the central focus for detection in any vision screening programme, primarily because it is not easily identified by the patient, their family, or through conventional healthcare means. In a similar vein, we aim to investigate whether this sometimes holds true for bilateral visual impairment (BVI) linked to refractive error. The overarching aim of this study was to therefore to determine the prevalence of presenting BVI associated with refractive error, detected via the Scottish preschool screening programme.

## Materials subjects and methods

The See4School programme is a comprehensive population screening programme administered by orthoptists in all mainland health boards (HB) across Scotland. The screening employs two widely recognised crowded letter tests: the Keeler LogMAR (KL) (Keeler, Windsor, UK), with a pass mark of ≤0.200logMAR and the Sonksen LogMAR (SL) (Haag-Streit, Harlow, UK), with a pass mark of ≤0.100logMAR [20]. For children who are unable to complete the letter test due to poor concentration or cognitive ability, the Kay Picture crowded (KPC) vision test (Kay Pictures Ltd, Herts, UK) is utilised with a pass mark of ≤0.100logMAR [21–23]. Children with refractive correction are assessed with their spectacle prescription and if they meet the predefined screening criteria, they are considered to have passed the test. Those who do not meet the criteria, are referred into the Hospital Eye Service (HES) for further diagnostic tests. The screening programme also incorporates additional tests, such as cover test (at 33 cm and 6 m), convergence, ocular movements, prism reflex test, and a basic stereo test.

The pre-school screening year within NHS Scotland spans from mid-August to late July, encompassing children ranging in age from 3 years 6 months to 5 years and 5 months, depending on their testing period. All screening results are recorded on a national form. Once completed, this form is sent to the HB’s child health (CH) department for input into the national information services division (ISD) database, within National Services for Scotland (NSS).

Children are referred from the screening programme, based on a number of pre-defined criteria, including failure in vision for the right eye, the left eye or both eyes, failure in orthoptic components or an inability to complete the test. Depending on availability of services within each HB, children will be reviewed by either the community optometrist, hospital optometrist or an ophthalmologist. Each HB has the same diagnostic pathway and spectacle prescription guidelines. The outcomes of the referral data are collected on an excel spreadsheet by each HB which is then anonymised before being merged into a national database. All children referred with reduced vision, proceed through the diagnostic pathway, which involves a comprehensive sight test. This sight test includes cycloplegic refraction, which is carried out using retinoscopy after the bilateral administration of cyclopentolate hydrochloride 1%. The cycloplegic agent helps to paralyse accommodation and obtain accurate measurements of refractive error. In cases where the initial cycloplegic effect is not sufficient to fully paralyse accommodation, repeat doses of cyclopentolate hydrochloride 1% or alternative medications such as atropine sulphate 1% may be administered during a separate appointment. This ensures that the children receive a thorough and accurate assessment of their refractive status, allowing for appropriate management and intervention if necessary.

**Fig. 1:**
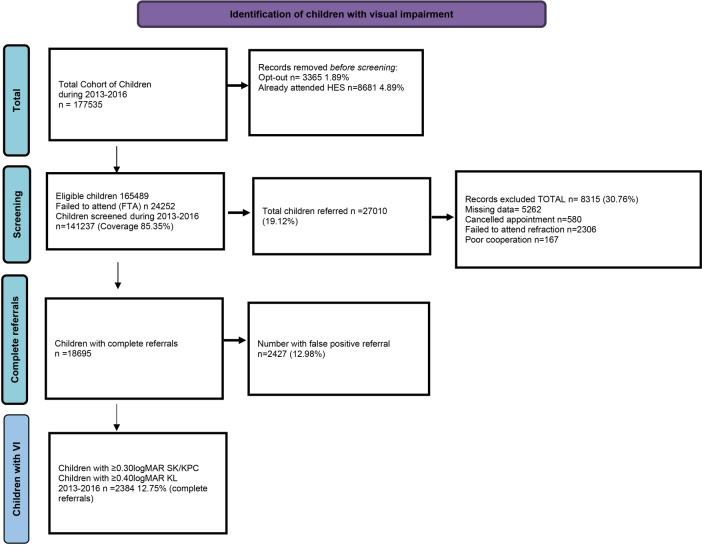
Flow diagram illustrating the numbers of children included in every stage of the research.

To enhance data quality, a systematic data cleaning procedure was implemented, as illustrated in Fig. 1. In the initial cleaning stage, specific data subsets were excluded, including children whose parents had opted out from the screening process, children already attending HES and those who failed to attend their vision screening appointment. In the subsequent cleaning stage, records with missing data, cancelled or incomplete sight test components and those pertaining to children with poor concentration were removed. Significant refractive error was defined as myopia or hypermetropia ≥ 1.00 DS; astigmatism ≥1.00 DC and/or anisometropia (either spherical or cylindrical) ≥ 1.00D [24, 25]. Children without significant refractive error and lacking any follow-up appointments were classified as false positive referrals. During this final stage of cleaning, a total of 2427 (12.98%) children with a false positive referral were excluded. After conducting the full sight test and eliminating false positive cases, the children were categorised as either having normal vision or those with visual impairment. The WHO-defined categories for visual impairment were applied to the vision or visual acuity results obtained from three different vision tests, as presented in Table 1 [26]. Mild visual impairment is normally classified as worse than 6/12 which equates to >0.30 logMAR. In Scotland the SCL and KPC have different pass/referral criteria compared against the KL test. This disparity reflects variations in testability of different vision tests in children at this age. Consequently, the visual impairment categories varied across different tests, as detailed in Table 1. The largest letter size on both the SL and KL test is 0.8 (6/36 equivalent). For the purpose of data analysis, the cut off for the severe category was set to 0.900 or worse for all three tests. For instance, if a child was unable to identify any of the 0.8 letters, on SL and KL at 3 m, this would be classified under the severe VI category. In cases where a child fell into different categories for each eye, they were assigned to the category corresponding the eye with better vision.

**Table 1:** Breakdown of vision tests in relation to Snellen VA, as defined by the WHO * Original version of the KPC test (4 pictures).

Level of Visual impairment Snellen Visual Acuity Scale as defined by WHO	Vision Test and LogMAR value
**Mild** Worse than 6/12 (0.300) to 6/18 (0.477)	SCL/KPC* 0.300 to 0.475
	KL 0.400 to 0.575
**Moderate** Worse than 6/18 (0.477) to 6/60 (1.00)	SKL/KPC 0.500 to 0.875
	KL 0.600 to 0.875
**Severe** Worse than 6/60 (1.00) to 3/60 (1.30)	SCL/KPC 0.900 or worse
	KL Worse 0.900 or worse

## Results

During the three-year period from 2013–2016, a total of 177,535 children were due to receive their See4School screening. Initial inspection of the data revealed that 8681 were already attending the hospital eye service, and 3365 parents or guardians had opted-out of the programme. The remaining 165,489 children were therefore eligible to have their vision screening assessment. In total 141,237 received their screening, meaning the service achieved an overall coverage of 85.35%. Among the 141,237 children who received screening, 27,010 (19.12%) failed and were referred for a full sight test. Of the children who failed their vision screening 408 (1.51%) were screened wearing their spectacle correction (Table 2).

**Table 2:** Numbers of children Screened with spectacle correction and the numbers that passed and failed.

Year	Children Screened with spectacles	Failed/Referred	Pass
2013–2014	313	109	204
2014–2015	394	143	251
2015–2016	425	156	269
**TOTAL**	1132	408	724

A total of 8315 (30.76%) of records were then excluded due to missing data, cancelled/missed appointments, or poor cooperation. In total there were 18,695 complete referral records were available for analysis. The overall false positive rate for the cohort was 12.98% (*n* = 2427). Among the true positive children with complete referral records, 12.75% (*n* = 2384) exhibited visual acuity (VA) of ≥ 0.3logMAR in both eyes

In the referral group with a complete data set (*n* = 18,695) (Table 3) the prevalence of children with mild, moderate and severe BVI varied between 1.21% (1.12–1.32) and 1.48% (1.37–1.59); 0.26 (0.21–0.31) and 0.44% (0.38–0.50); 0-0 (0–0.02) and 0.02% (0.01–0.03) respectively. Total BVI therefore ranged between 1.47% (1.37–1.59) and 1.93% (1.81–2.06).

**Table 3:** Percentage (%) of children with bilateral visual impairment ≥0.30logMAR (VI) and their confidence intervals (CI) for all children referred with a complete data set.

	Year 2013/14	Year 2014/15	Year 2015/16
Mild VI %	1.21	1.34	1.48
Mild VI CI	1.12–1.32	1.23–1.44	1.37–1.59
Moderate VI %	0.26	0.32	0.44
Moderate VI CI	0.21–0.31	0.27–0.38	0.38–0.50
Severe VI %	0.00	0.01	0.02
Severe VI CI	0–0.02	0–0.02	0.01–0.03
TOTAL VI %	1.47	1.66	1.93
TOTAL VI CI	1.37–1.59	1.55–1.78	1.81–2.06

A significant proportion of children who failed their vision screening test, amounting to over 30%, were initially excluded from the final data analysis due to missing or incomplete data (Fig. 1). This exclusion posed a challenge as it was likely that many of these children also have had BVI. To overcome this limitation and provide a more comprehensive understanding of the prevalence of presenting BVI, an estimation approach was adopted. It was assumed that children with missing or incomplete records would follow the same pattern as those with complete records, exhibiting similar numbers of false positive records and the same numbers of mild, moderate and severe BVI.

To account for this issue, the prevalence of BVI was therefore categorised in two ways. Firstly, using only those records with a completed cleaned data set, which inherently underestimates the prevalence. Secondly, by including the children with a missing or incomplete data set and assuming that they would exhibit the same pattern as those with a complete data. After removing the anticipated percentage of false positives from the incomplete data sets, we forecasted that the remaining data would produce similar percentages of mild, moderate and severe BVI (as those with a complete data set) and therefore adjusted the figures accordingly. By employing this estimation method, the aim was to mitigate the impact of the missing or incomplete data and provide a more accurate representation of the prevalence of presenting BVI.

In Table 4 the percentage prevalence and CI for children referred with a complete data set plus the forecasted numbers from the missing data is reported. The prevalence of children with mild, moderate and severe BVI varied between 1.58% (1.47–1.70) and 1.9% (1.73–1.98); 0.33 (0.28–0.39) and 0.55% (0.49–0.62); 0.01% (0–0.02) and 0.02% (0.01–0.04) respectively. Total BVI therefore ranged between 1.92% (1.80–2.05) and 2.42% (2.29–2.57).

**Table 4:** Percentage (%) of children with bilateral visual impairment ≥ 0.30logMAR (VI) and their confidence intervals (CI) for all children referred with a complete data set plus the forecasted numbers for the missing data, across three years.

	Year 2013/14	Year 2014/15	Year 2015/16
Mild VI %	1.58	1.66	1.9
Mild VI CI	1.47–1.70	1.55–1.78	1.73–1.98
Moderate VI %	0.33	0.4	0.55
Moderate VI CI	0.28–0.39	0.34–0.46	0.49–0.62
Severe VI %	0.01	0.01	0.02
Severe VI CI	0–0.02	0–0.02	0.01–0.04
TOTAL VI %	1.92	2.1	2.42
TOTAL VI CI	1.80–2.05	1.94–2.20	2.29–2.57

## Discussion

Children already receiving eye care from the hospital eye service were excluded from the entire data analysis. This data therefore does not represent the total prevalence of BVI associated with refractive error. Instead, the data reflects the presenting levels of BVI associated with refractive error, in children who were previously undetected or unsuccessfully treated by community optometrists. Following their exclusion, the prevalence figures for BVI associated with refractive error remained high, ranging between 1.92 to 2.42% across all three the years. Among the children that were screened and failed, only 408 (1.51%) were likely under the supervision of a community optometrist, which was evident from the fact that these children presented with their spectacle correction. Present results therefore suggest that these children had not been successfully treated, as they all exhibited BVI despite wearing corrective lenses. Findings indicate that in Scotland there are thousands of preschool children every year, primarily with undetected BVI which is associated with refractive error.

One limitation of this study is the potential underestimation of the numbers of children with BVI detected via the screening programme, stemming from several factors. Firstly, the study did not apply a uniform criterion of 0.30 logMAR across all three vision tests used. Had this ‘worse than or equal to 0.30 logMAR’ criterion been used throughout the screening programme; it is likely that the prevalence figures would have been notably higher. However, our approach was necessitated by Scotland’s utilisation of distinct pass criteria for the three vision tests, accommodating variations in testability rates among children of this age. Furthermore, adopting more stringent visual acuity cut-offs for distance vision impairment, as commonly practiced in higher-income countries, could also have significantly increased the prevalence [1].

Children who miss scheduled appointments are likely to belong to households facing heightened obstacles to accessing eye care services [27]. Those hailing from socioeconomically disadvantaged backgrounds are prone to lower attendance rates at screening and have higher failure rates in screening due to an increased prevalence of certain childhood eye issues such as hypermetropia, esotropia, and amblyopia [12, 28]. Additionally, the 0.62% of children who failed screening and demonstrated poor cooperation during screening are also more likely to have significant vision problems compared to those with normal cooperation [29]. The assumption that these children will have similar prevalences of BVI to the sample analysed will likely underestimate the numbers of undetected and unsuccessfully treated children with BVI. The methodological constraints outlined here, which were chosen to address missing and unknown data, should all be recognised as reducing the reported prevalence of bilateral visual impairment. These figures, therefore, do not represent the total prevalence of bilateral visual impairment or the prevalence figures for all the visual defects that a vision screening programme detects. Instead, they represent the minimum levels of bilateral visual impairment associated with refractive error in children who were previously undetected or successfully treated.

Visual impairment has significant functional consequences in children, with the potential to disrupt normal development and impact daily life [30, 31]. Individuals with visual impairment face difficulties in perceiving people, objects, and print, leading to challenges in participating in activities that require good visual discrimination. Limited spatial awareness hampers engagement in physical activities and hinders full interaction with peers and the environment [31]. Visual impairment also disturbs a child’s social interaction and emotional wellbeing as they may experience difficulties in recognising facial expressions, making eye contact or interpreting non-verbal clues [32]. These challenges can lead to feelings of social isolation, behavioural problems and low self-esteem [32, 33].

A growing body of research highlights the negative impact of reduced vision on a child’s academic performance [11, 15, 17–19]. Bruce et al. [11] found that for every line reduction of visual acuity lowered their literacy score by 2.42 points. A separate paper published by Bruce et al. in 2018 [19], reiterated a link between poorer visual acuity and lower literacy scores and subsequently led to the Glasses in Classes project [34], which found that the literacy attainment gap of the children narrowed by approximately half when glasses were worn regularly. The project involved both the families and the teachers supporting the wearing of refractive correction in school. The scheme has been so successful, that it has been given additional funding to include a further five disadvantaged areas in England, as of September 2022 [35]. These results are unsurprising considering previous research which has documented that correcting refractive errors with appropriate spectacles is among the most cost-effective interventions in eye health care [36].

In the United States, Medicaid, a government programme for low-income families, consider children’s vision to be an essential component of their early screening programme. It is recognised worldwide, that there is higher incidence of visual problems in low-income families [12, 37]. The Scottish Government has made significant investment through the Scottish Attainment Challenge [38]. The goal is to improve literacy outcomes and reduce the attainment gap for children impacted by poverty. Vision screening needs to be prioritised as having a key role in this education goal. Recent literature indicates that correctable BVI is increasing post-covid [39–41]. Unfortunately there are Integrated Care Group’s (ICG’s) in Public Health England (PHE) that have never re-established vision screening post pandemic and others that have never had screening in place. It is concerning that vision screening is still a post-code lottery in the UK, with children in Scotland receiving a comprehensive orthoptic delivered service [24, 42] while other areas of the UK have no vision screening in place.

Vision screening in Scotland is currently a recommendation rather than a fully commissioned, mandated screening programme. Following the pandemic many Scottish HB’s are finding it challenging to maintain the high coverage that was achieved pre-pandemic. Delays caused by COVID 19 pandemic have been difficult to recover from, with some health boards screening significantly later. This delays detection and treatment of visual problems meaning children with preventable BVI, will be struggling through the first year of school before having their visual issue detected and glasses prescribed/treatment started.

The Royal National Institute for the blind (RNIB) published Key Statistics about Sight Loss in 2021 [43] predicting that by 2050 the number of people with sight loss in the UK will double to over four million. Of the top seven causes of visual impairment, 39% was due to uncorrected refractive error. With additional funding the See4School programme in Scotland could achieve higher coverage by targeting areas of poverty, and ensure robust referral pathways are in place, to ensure children are getting access to glasses and are wearing them. The incidence of eye conditions such as diabetic retinopathy and glaucoma [44, 45] is predicted to increase rapidly over the coming years, pre-school vision screening is therefore vital for early detection and treatment of correctable BVI.

## Conclusions

One of the limitations of this study was incomplete data collection, particularly in relation to missing refraction results, which was most pronounced amongst the referral to community optometry group. Test variability made analysis of the visual acuity cut-offs challenging. Electronic forms and accredited pathways for referrals going to community optometry would allow more complete data collection which would reduce these variables dramatically. In this large population-based screening cohort, mild moderate and severe correctable BVI associated with refractive error often goes undetected by parents and carers with up to 2.42% (2.29–2.57) of children having poorer than driving standard vision (6/12) in their pre-school year. The See4School pre-school vision screening programme in NHS Scotland has demonstrated effectiveness in detecting correctable BVI, with an acceptable false positive rate (12.98%). To optimise educational attainment and preserve the vision of future generations it is imperative to prioritise the prevention of treatable childhood vision problems when commissioning services.

## Summary

### What was known before

- Until now the prevalence of presenting bilateral visual impairment associated with refractive error, from the pre-school vision screening programme in NHS Scotland was unknown.

### What this study adds

- This paper quantifies the prevalence of presenting bilateral vision impairment associated with refractive error in pre-school children.
- It is estimated that up to 2.42% (2.29–2.57) of children living Scotland have poorer than driving standard of vision (6/12) in their pre-school year.
- Reduced vision has the potential to impact a child’s day-to-day life including their future educational, health and social outcomes.
- Prioritising vision screening in Scotland is crucial for optimising preventative healthcare and reducing inequalities from an early age.
